# Individualized Treatment Effects of a Digital Smoking Cessation Intervention Among Individuals Looking Online for Help: Secondary Analysis of a Randomized Controlled Trial

**DOI:** 10.2196/63578

**Published:** 2026-02-11

**Authors:** Joel Crawford, Jenny Blomqvist, Katarina Ulfsdotter Gunnarsson, Preben Bendtsen, Marcus Bendtsen

**Affiliations:** 1 Department of Health, Medicine and Caring Services, Linköping University, Linköping, 581 83, Sweden, 46 13 28 10 00; 2 Department of Medical Specialist, Motala Hospital, Motala, Sweden

**Keywords:** individualized treatment effects, randomized controlled trial, text message–based smoking cessation intervention, smoking cessation, smoking, Sweden, behavior change, text messages, smoker

## Abstract

**Background:**

Smoking is a leading cause of mortality and morbidity worldwide. Efforts to reduce smoking prevalence have used SMS text message–based interventions, which typically send participants a series of short, informational, motivational, and practical messages over a set period. Evidence highlights the efficacy of using this approach to support smoking cessation, with such trials typically reporting the average treatment effects, in which causal inference is made regarding the average effect of a treatment on a heterogeneous sample. Nonetheless, using this approach to assessing treatment effects means we are unable to account for individual factors that impact the effectiveness of a treatment on outcomes, such as age, gender, and genetics.

**Objective:**

This study aimed to estimate the individualized effects of an SMS text message–based smoking cessation intervention to ascertain which individuals benefited the most and least during an effectiveness trial.

**Methods:**

Data from a randomized controlled trial including 1012 adults from the Swedish general population were used. The trial assessed the effects of an SMS text message–based intervention, NEXit (Nicotine Exit), that aimed to change behavior by increasing the importance of change, boosting knowledge on how to change, and instilling confidence for change. Outcomes assessed in the trial were prolonged abstinence and point prevalence of smoking cessation. Individualized treatment effects were modeled using baseline factors (demographics, psychosocial variables, and past behavior) to study who benefited the most and least from the intervention.

**Results:**

For prolonged abstinence, there was evidence of heterogeneous effects, with those benefiting the most from NEXit being older adults, female participants, individuals with high confidence in their ability to quit, and those who believed that quitting was important. For point prevalence abstinence, older individuals and those reporting high confidence in the ability to quit, the importance of quitting, and knowledge for change benefited the most. For both outcomes, individuals who reported smoking for a longer duration and smoking more at baseline benefited less.

**Conclusions:**

The results demonstrate how individuals respond differently to an SMS text message–based smoking cessation intervention. This provides an insight into who benefits the most and least from the intervention in terms of demographics, baseline characteristics, and behaviors. The study highlights which individuals need to be specifically targeted and/or have content developed to suit their individual needs to further reduce the prevalence of smoking.

## Introduction

Smoking remains a leading cause of morbidity and mortality across the globe, with recent figures highlighting approximately 8 million deaths worldwide and 200 million disability-adjusted life years attributed to smoking [1,2]. In Sweden, the prevalence of smoking within the general population has consistently decreased over the last 2 decades, with the Public Health Agency of Sweden reporting that prevalence rates dropped to 6% for women and 5% for men in 2022 [3]. Despite this, smoking remains a leading cause of mortality and morbidity within Sweden [4] and is more prevalent in groups with lower socioeconomic status [5]. To stem the associated morbidity and mortality and reduce health inequalities, effective interventions aimed at reducing the prevalence of smoking within the general population are needed.

Current efforts to reduce smoking prevalence have used SMS text message–based interventions, which typically comprised a series of short messages sent to participants over a designated time period (usually 2-3 mo) [6,7]. The aim of these interventions is to provide motivation to quit, support for quitting, and reinforcement of the decision to quit [7]. A series of meta-analyses have highlighted the effectiveness of SMS text message–based smoking cessation interventions, and all concluded positive effects were observed for both self-reported and biochemically verified abstinence [6-10].

In Sweden, a recent randomized controlled trial (RCT) estimated the effectiveness of receiving a SMS text message–based smoking cessation intervention against existing public support (ie, referral to the national Quitline and website, slutarokalinjen.se) [11]. The trial targeted the general population using the NEXit (Nicotine Exit) protocol [12]. Effect estimates were made at 3 and 6 months post randomization for both prolonged abstinence (ie, the Russell standard definition of not having smoked more than 5 cigarettes in the past 8 wk) and point prevalence of abstinence. At 3 months post randomization, the odds ratio (OR) of prolonged abstinence was 2.15 (95% compatibility interval [CoI] 1.51-3.06), and at 6 months, the OR of prolonged abstinence was 2.38 (95% CoI 1.62-3.57). For point prevalence of abstinence, the OR at 3 months was 1.70 (95% CoI 1.18-2.44), and at 6 months, it was 1.49 (95% CoI 1.03-2.14).

While the findings suggest the intervention is effective for smoking cessation, the results of the RCT were assessed at the group level, meaning we are unable to account for how individual differences impact outcomes. This is pertinent, as not everyone in the intervention group successfully quit smoking, meaning that some individuals benefited more from the intervention than others. Individual differences in terms of fixed (eg, age, ethnicity, gender) and modifiable (eg, socioeconomic status, psychological) health factors have been shown to impact abstinence rates [13-16], which can potentially account for why some individuals benefit more from treatment than others.

Currently, Sweden has a national target of becoming a “smoke-free” state (ie, <5% prevalence in the general population) [17]. To achieve this target, smoking cessation support such as the NEXit protocol needs to become specifically targeted to those who continue to smoke tobacco despite policy measures and interventions. The aim of this study is to ascertain who benefited the most and least in the recent NEXit trial [11] through a secondary analysis of the group-level data, which estimated the average treatment effect of NEXit in the Swedish general population. We aim to achieve this by assessing how baseline factors influence outcomes of the trial at the individual level.

## Methods

### Study Design

The current study investigated the individualized effects of the NEXit protocol using data from a 2-arm, parallel-group (1:1) RCT. The trial was registered in the ISRCTN registry on December 3, 2020 (ISRCTN13455271), and a protocol and an analysis plan were published prospectively [12].

### Participants and Settings

A total of 1012 participants from the Swedish general public were recruited from 2 settings: (1) online advertisements, in which individuals clicked on a link that took them to an information page with instructions for signing up, and (2) primary health care units in southern Sweden, which used printed media (leaflets and posters) containing instructions for signing up. Individuals were required to be aged 18 years or older, have access to a mobile phone, and smoke at least one cigarette per week to participate in the trial. Regardless of medium, those interested in participating sent an SMS text message to a dedicated number and received a reply within 5 minutes with a link to the study information and the informed consent form. After providing consent, participants were asked to complete a baseline survey, which was used to assess eligibility. Immediately afterward, eligible participants were randomly assigned to 1 of 2 study groups.

### Ethical Considerations

The trial received ethical approval from the Swedish Ethical Review Authority on June 16, 2020 (Dnr 2020‐01427). Participants provided informed consent prior to the commencement of the main trial, which included consent for their deidentified data to be analyzed and published. Trial data are deidentified, and participants in the main trial did not receive compensation.

### Interventions

Both the control and intervention groups were offered treatment as usual and were thus not prohibited from using publicly available smoking cessation support aids. Treatment as usual was referral to a national smoking cessation helpline and website; those recruited from primary care units also had the option of meeting with a nurse or smoking cessation counselor. Additionally, participants in the intervention group were given access to the NEXit SMS text messaging intervention. The intervention had 2 fairly similar versions: a general version and a version for those undergoing elective surgery (within 3 mo of signing up). The intervention consists of a 1- to 4-week motivational phase in which participants were encouraged to set a date on which to quit, followed by a 12-week program consisting of 157 cessation support messages. Messages were typically informational and encouraging, and they provided tips and techniques for creating a smoke-free environment and lifestyle. These included distraction techniques, tips to avoid weight gain, tips to cope with cravings, tips on how to avoid triggers, tips to self-regulate, and practical tips to help restructure the physical environment. The design of the messages was informed by intervention mapping [18] and the behavior change wheel [19], with various behavior change techniques being utilized, including 1.2 problem solving, 1.4 action planning, 5.1 information about health consequences, 5.2 salience of consequences, 8.2 behavioral substitution, 8.4 habit reversal, 9.1 credible source, 10.9 self-reward, 12 restricting the physical environment, 12.3 avoidance to cues, and 12.4 distraction. Messages were sent daily to participants, and the elective surgery version contained additional messages regarding the impact of smoking on surgery and recovery. Participants could also text and request additional support if they had relapsed or were experiencing cravings.

### Outcomes and Measures

The primary outcomes of the trial were assessed at 3 and 6 months post randomization:

1. Prolonged abstinence, using the Russell standard definition of not having smoked more than 5 cigarettes in the past 8 weeks at the 3-month follow-up and in the past 5 months at the 6-month follow-up
2. Point prevalence, defined as not smoking any cigarettes during the past 4 weeks at both follow-up intervals

Baseline factors assessed in this secondary analysis of individualized effects include factors that are associated with cessation, including demographics (age and gender) and smoking behaviors (number of years smoking, amount of cigarettes smoked per week, use of snus [akin to snuff tobacco], number of past quit attempts, and nicotine dependence score using the Fagerström scale, which uses 6 items to assess intensity of physical addiction, including quantity and frequency of smoking) [13,20-24]. The recruitment setting (online or primary care unit) and whether participants were undergoing elective surgery were also assessed. Psychosocial variables (importance of quitting, confidence in the ability to quit, and knowledge on how to quit) were assessed. These factors are reflective of the behavioral and control beliefs that drive behavior, as outlined in expectancy-value models of health [25,26]. Extant literature highlights that the demographic factors (age and gender) are associated with other baseline factors used in our modeling [13,20-22].

### Randomization and Blinding

Randomization was completed using a computer-generated random sequence, stratified according to which version of the intervention was appropriate (general or surgery). Block randomization, using block sizes of 2 and 4, ensured equal numbers of participants were allocated to the groups. Participants received notification of group allocation via SMS text message. Research personnel were blinded to allocation, and all procedures of the study were automated, except follow-up calls to nonresponders. For a full description of all study procedures, see Bendtsen et al [12].

### Statistical Model

The observed outcome (*Y*) was modeled as a function of group allocation (*G*) and baseline variables (*X*) to estimate the individualized treatment effect $\delta \left(x_{i}\right)=P\left(Y_{i}|G=1,X=x_{i}\right)-P\left(Y_{i}=|G=0,X=x_{i}\right)$, following the approach by Hoogland et al [27]. Here, $\delta \left(x_{i}\right)$ represents the difference between the predicted probability of smoking cessation ($Y_{i}$) for an individual (i) with baseline factors $x_{i}$, if they had access to the intervention (G=1) versus if they did not (G=0). Individualized treatment effects were modeled for each participant as a function of baseline factors to study which individuals had the most and least benefit from the intervention.

Smoking cessation outcomes at 3 and 6 months post randomization were modeled using multilevel logistic regression with a time-by-group interaction term. Available data were used to estimate the model and predict outcomes for all randomized participants. The models included participant-level adaptive intercepts, covariates for baseline factors, and interactions between baseline factors and group allocation. Bayesian inference was used to estimate the parameters of the models [28]. To promote parsimonious models, priors that shrunk estimates toward the null for baseline factor interaction coefficients were used, specifically Cauchy priors centered at the null with a standard normal hyperprior for the scale parameter [28]. We used Student *t* test priors centered at 0, with 3 *df* and a scale of 2.5 for noninteraction coefficients and standard normal priors for adaptive and fixed intercepts. Individualized treatment effects were calculated from posterior predictive draws, resulting in posterior distributions over treatment effects for each individual. Medians of these posterior distributions were used as point estimates of $\delta \left(x_{i}\right)$.

Linear regression was used to study associations between standardized estimated individualized effects and baseline factors. Student *t* test priors centered at 0 with 3 *df* and a scale of 2.5 for all coefficients in these models. Medians of posterior distributions of covariates were used as point estimates and created 95% CoI using the 2.5 and 97.5 percentiles of the posterior distributions. Estimation was done using CmdStan version 2.33.0 (Stan Project), which is a shell interface for Stan [29]. Plots were generated using the ggplot2 library in R.

## Results

### Participant Retention and Missing Data

**Figure 1.**
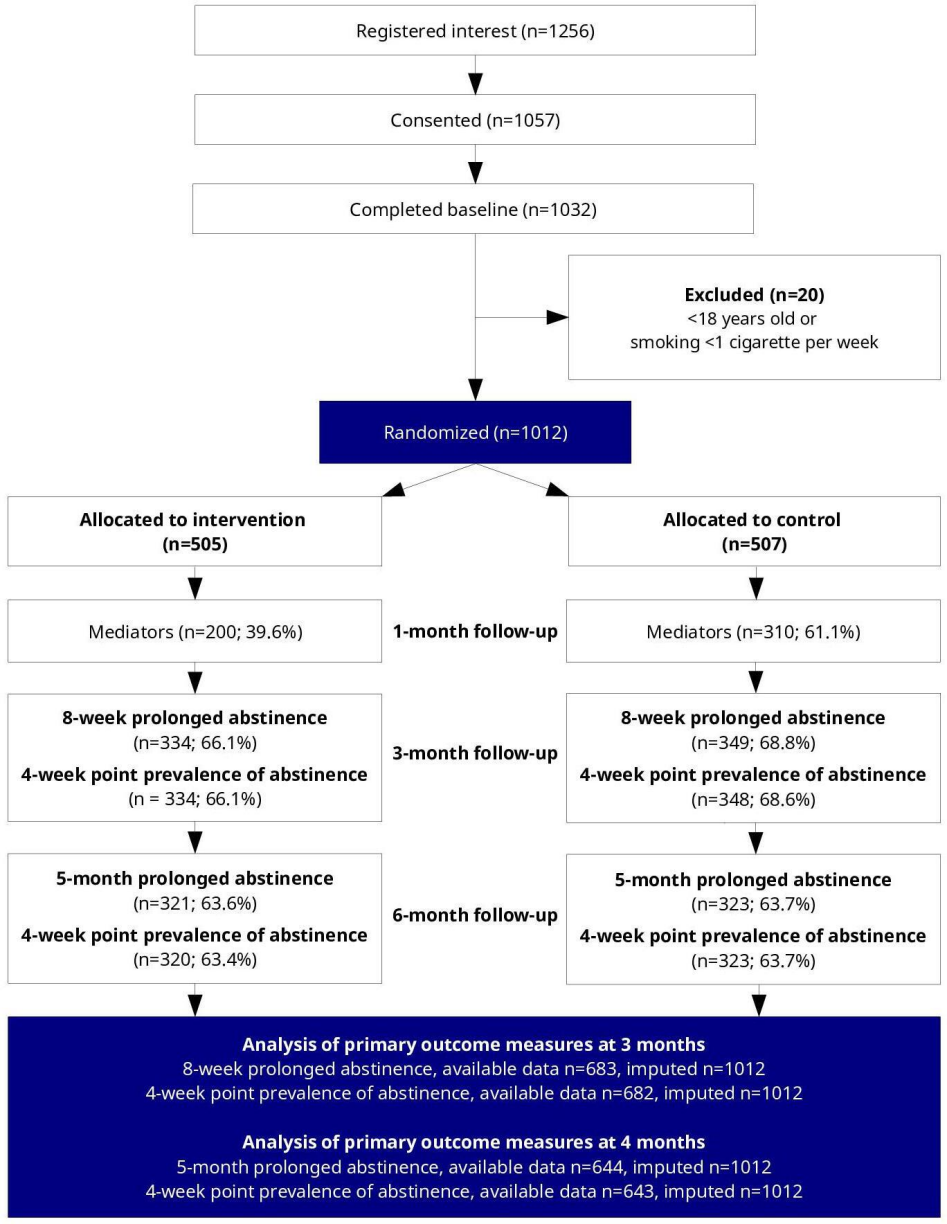
CONSORT (Consolidated Standards of Reporting Trials) flowchart.

Table 1 presents baseline factors for the 1012 participants recruited to the trial. At the 3-month follow-up interval, data were available for 66% of participants in the intervention group and 69% of participants in the control group. The corresponding proportions for the 6-month interval were 64% for both groups. These data were used to estimate the multilevel logistic regression models, which were subsequently used to estimate individualized treatment effects for all randomized participants. As reported in the primary findings of the trial [11], there was evidence that older participants were more likely to respond to follow-up at the 6-month interval, but no other associations were found between response and baseline factors. The CONSORT (Consolidated Standards of Reporting Trials) flowchart and checklist are presented in Figure 1 and Checklist 1.

**Table 1.** Baseline factors of participants.

	Total (N=1012)	Intervention (n=505)	Control (n=507)
Woman^a^, n (%)	820 (81)	406 (80.4)	414 (81.7)
Age (years), mean (SD)	45.4 (14.0)	45 (13.9)	45.7 (14.1)
Years of smoking, mean (SD)	25.3 (14.6)	24.7 (14.3)	25.9 (14.9)
Daily smokers (vs weekly smokers), n (%)	981 (96.9)	489 (96.8)	492 (97)
Cigarettes smoked per week, mean (SD)	101 (46.2)	101.4 (47.3)	100.6 (45.1)
Use of snus^b^, n (%)			
Daily	63 (6.2)	27 (5.3)	36 (7.1)
Weekly or monthly	90 (8.9)	45 (8.9)	45 (8.9)
No	859 (84.9)	433 (85.7)	426 (84)
Fagerström test for nicotine dependence, mean (SD)	5 (2.2)	5 (2.2)	5 (2.2)
Quit attempts^c^, mean (SD)	7.2 (13.7)	7.0 (12.7)	7.5 (14.6)
Cessation counseling experience, n (%)			
Yes, now	32 (3.2)	13 (2.6)	19 (3.7)
Yes	192 (19)	95 (18.8)	97 (19.1)
No	788 (77.9)	397 (78.6)	391 (77.1)
Used quit smoking helpline, n (%)	13.6 (138)	14.5 (73)	12.8 (65)
Importance of quitting^d^, mean (SD)	9.4 (1.3)	9.4 (1.3)	9.5 (1.2)
Confidence in ability to quit^d^, mean (SD)	6.2 (2.5)	6.3 (2.5)	6.2 (2.6)
Knowledge of how to quit^d^, mean (SD)	5.5 (2.6)	5.5 (2.7)	5.5 (2.5)
Elective surgery^e^, n (%)	63 (6.2)	31 (6.1)	32 (6.3)

a The baseline questionnaire included a category “Other”; however, it was not chosen by any participant.

b Snus is a moist oral tobacco product, which is common in Sweden, sometimes translated as snuff*.*

c Participants were asked about the lifetime number of quit attempts.

d Three single-item measures were used to assess the importance, confidence, and know-how regarding smoking cessation. Responses ranged from 0 to 10, with 10 representing the highest agreement (ie, very important, very confident, very knowledgeable). The same items were used at follow-up as hypothesized mediators of effects.

e Participants self-reporting that they had planned elective surgery received additional intervention materials regarding smoking and surgery.

### Baseline Factors and Individualized Treatment Effects

**Figure 2.**
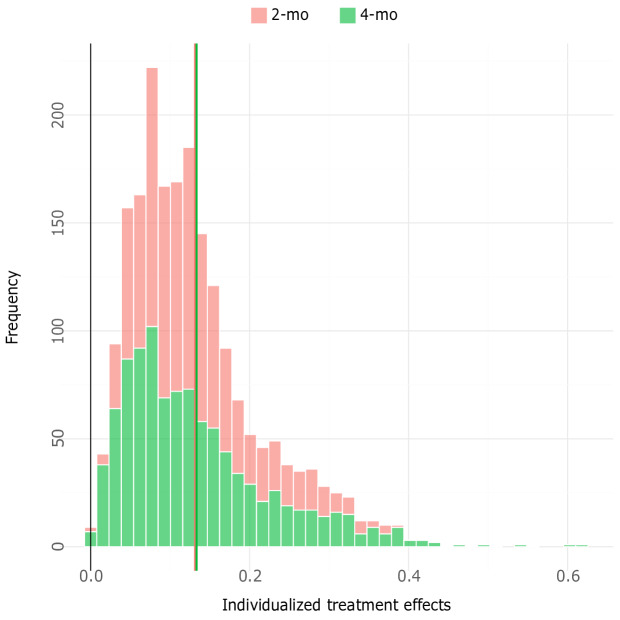
Distribution of effects for prolonged abstinence.

**Figure 3.**
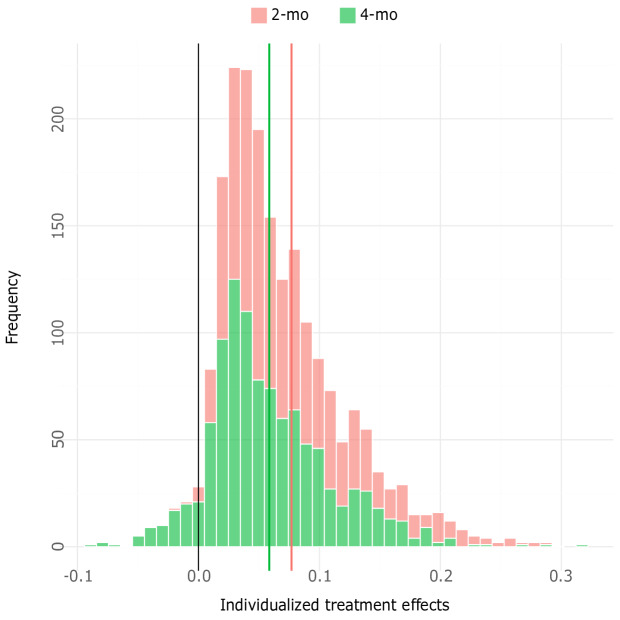
Distribution of effects for point prevalence abstinence.

In Figure 2, the distribution of effects for 8-week or 5-month abstinence is presented for both the 3- and 6-month follow-up intervals, and Figure 3 provides the distribution of effects for point prevalence abstinence for both follow-ups. The figures show the effects of NEXit at the individual level, expressed as differences in probability (percentage points) of smoking cessation between having received the intervention and the control. From the figures, it is evident that most participants were predicted to benefit from the intervention; however, more so in terms of prolonged abstinence at the 6-month follow-up interval. The mean individualized effect for point prevalence at the 3-month interval was 3.9 (SD 5.3) percentage points and 2.7 (SD 3.9) percentage points at the 6-month interval. For prolonged abstinence, the mean individualized effect was 7.5 (SD 8.5) percentage points at 3 months and 7.8 (SD 10.7) percentage points at 6 months. This indicates that the intervention was most effective in producing prolonged abstinence, but more importantly, that individualized effects were overdispersed and showed heterogeneity of effects in the population.

### Prolonged Abstinence

In Table 2, associations between baseline factors and standardized individualized treatment effects on prolonged abstinence are presented for both follow-up intervals. For both time points (3 and 6 mo), there was marked evidence for positive associations between individualized effects and gender, age, importance, and confidence. Meanwhile, there was marked evidence for negative associations between individualized effect and number of cigarettes smoked at baseline and years of smoking. Associations between individualized effects and recruitment setting, and past professional cessation support, were found for the 6-month follow-up period.

**Table 2.** Baseline factors and standardized individualized effects on prolonged abstinence.

	8 wk		5 mo	
	Est^a^, OR^b^ (95% CoI^c^)	Prob^d^ (%)	Est^a^, OR (95% CoI)	Prob^d^ (%)
Man vs woman	0.34 (0.19 to 0.49)	>99.9	0.18 (0.03 to 0.32)	99.1
Age	0.02 (0.01 to 0.03)	>99.9	0.02 (0.02 to 0.03)	>99.9
Importance	0.09 (0.05 to 0.14)	>99.9	0.1 (0.05 to 0.14)	>99.9
Confidence	0.05 (0.03 to 0.08)	>99.9	0.06 (0.03 to 0.08)	>99.9
Know-how	0.01 (-0.01 to 0.04)	83.7	0.02 (0.0 to 0.05)	97.6
Cigarettes smoked	–0.01 (–0.01 to –0.01)	>99.9	–0.01 (–0.01 to –0.004)	>99.9
Years smoking	–0.01 (–0.02 to –0.004)	99.8	–0.02 (–0.03 to –0.01)	>99.9
Quit attempts	–0.01 (–0.01 to –0.004)	>99.9	0.01 (0.006 to 0.014)	>99.9
Snus, weekly or monthly vs daily	–0.08 (–0.37 to 0.21)	70.5	–0.11 (–0.40 to 0.18)	76.0
Snus, none vs daily	–0.11 (–0.35 to 0.12)	83.1	0.02 (–0.21 to 0.25)	56.1
Fagerström score	0.01 (–0.02 to 0.04)	72.9	0.02 (–0.01 to 0.06)	89.4
Elective surgery	0.63 (0.40 to 0.86)	>99.9	0.50 (0.27 to 0.73)	>99.9
Online vs primary care	–0.09 (–0.37 to 0.20)	71.7	–0.46 (–0.74 to –0.17)	99.9
Quitline	–0.03 (–0.20 to 0.13)	64.2	0.14 (–0.03 to 0.30)	95.1
Past cessation support	–0.12 (–0.27 to 0.03)	93.5	–0.21 (–0.36 to –0.06)	99.7
Current cessation support	0.24 (–0.08 to 0.56)	92.6	0.17 (–0.15 to 0.48)	84.6

a Est: median of the posterior distribution of standardized individual effects with 95% CoIs.

b OR: odds ratio.

c CoI: compatibility interval.

d Prob: proportion of the posterior distribution above or below the null in the direction of the median.

### Point Prevalence Abstinence

In Table 3, associations between baseline factors and standardized individualized treatment effects on point prevalence are presented for both follow-up intervals. For both time points, there was marked evidence of positive associations between individualized effect and age, importance, confidence, know-how, and elective surgery at the 6-month interval. Negative associations between individualized effects and the number of cigarettes smoked at baseline and years of smoking were found.

**Table 3.** Baseline factors and standardized individualized effects on point prevalence.

	3 mo		6 mo	
	Est^a^, OR^b^ (95% CoI^c^)	Prob^d^ (%)	Est^a^, OR (95% CoI)	Prob^d^ (%)
Man vs Woman	0.05 (–0.10 to 0.20)	73.7	0.011 (–0.13 to 0.16)	55.7
Age	0.03 (0.02 to 0.03)	>99.9	0.02 (0.011 to 0.03)	>99.9
Importance	0.05 (0.01 to 0.10)	98.5	0.07 (0.03 to 0.12)	99.9
Confidence	0.05 (0.02 to 0.07)	>99.9	0.04 (0.02 to 0.07)	>99.9
Know-How	0.02 (–0.004 to 0.04)	94.4	0.02 (–0.001 to 0.05)	97.0
Cigarettes smoked	–0.01 (–0.01 to –0.01)	>99.9	–0.01 (–0.01 to –0.01)	>99.9
Years smoking	–0.02 (–0.03 to –0.02)	>99.9	–0.03 (–0.04 to –0.02)	>99.9
Quit attempts	–0.002 (–0.01 to 0.002)	84.6	0.002 (–0.002 to 0.01)	85.7
Snus, weekly or monthly vs daily	–0.03 (–0.32 to 0.26)	57.2	–0.43 (–0.71 to –0.14)	99.9
Snus, none vs daily	–0.12 (–0.35 to 0.11)	84.4	–0.13 (–0.91 to 0.65)	63.7
Fagerström score	–0.02 (–0.05 to 0.01)	86.6	0.01 (–0.02 to 0.04)	75.1
Elective surgery	0.20 (–0.03 to 0.43)	95.7	0.37 (0.15 to 0.60)	99.9
Online vs primary care	0.19 (–0.09 to 0.47)	90.4	0.01 (–0.27 to 0.29)	51.5
Quitline	0.09 (–0.08 to 0.25)	84	0.01 (–0.16 to 0.18)	53.9
Past cessation support	–0.04 (–0.19 to 0.11)	71.7	–0.04 (–0.19 to 0.11)	70.7
Current cessation support	–0.12 (–0.45 to 0.21)	76.1	–0.13 (–0.45 to 0.19)	78.6

a Est: Median of the posterior distribution of standardized individual effects with 95% compatibility intervals (CoI).

b OR: odds ratio.

c CoI: compatibility interval.

d Prob: Proportion of the posterior distribution above or below the null in the direction of the median.

### Interactions

**Figure 4.**
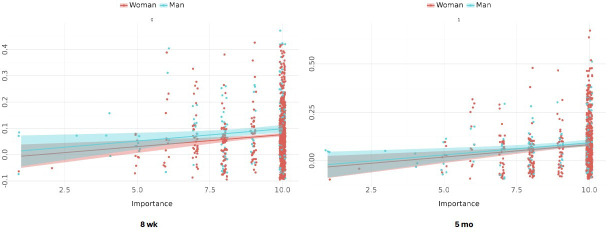
Visualization of interaction associations of gender and baseline importance to quit smoking on prolonged abstinence.

To assess whether the heterogeneous main associations for age and gender were qualified by interactions, models with added interaction terms were estimated. The only interaction for which there was marked evidence was between gender and importance to quit smoking, which was associated with individualized effects of the intervention on prolonged abstinence. This suggests that the main association of gender for this outcome was accounted for by its interaction with importance. Please see Table 4 for estimates of the interaction and Figure 4 for plots illustrating these interactions.

**Table 4.** Interaction associations of gender and baseline importance to quit smoking on prolonged abstinence.

	8 wk		5 mo	
	Est^a^, OR^b^ (95% CoI^c^)	Prob^d^ (%)	Est^a^, OR (95% CoI)	Prob^d^ (%)
Man vs woman	0.004 (–0.12 to 0.28)	56.6	0.0 (–0.22 to 0.18)	50
Importance	0.06 (0.011 to 0.11)	99.4	0.08 (0.03 to 0.13)	>99.9
Man vs woman × importance	0.03 (0.01 to 0.05)	98.4	0.02 (–0.004 to 0.05)	95.4

a Est: Median of the posterior distribution of standardized individual effects with 95% compatibility intervals (CoI).

b OR: odds ratio.

c CoI: compatibility interval.

d Prob: Proportion of the posterior distribution above or below the null in the direction of the median.

## Discussion

### Age

The current study assessed the individualized treatment effects of the recent NEXit SMS text message–based intervention [11]. We found an association between age and individualized effects (ie, as age increases beyond the mean value), with older participants benefiting more than younger participants for both prolonged and point prevalence abstinence. This finding contrasts with evidence comparing effects across trials, which highlights that effective interventions for the adult population in general have similar effects for younger adults [30]. This disparity may be a result of the content of the intervention, which specifically focused on support to restructure the physical environment rather than support for the social environment. This is important, as younger adults have a high prevalence of social smoking [20], which may account for this effect. Future interventions for this subsample could include aspects targeting social factors. Nonetheless, evidence also highlights that younger adults are more likely to initiate cessation compared to older adults and smoke less heavily [13,20]; therefore, the results of the current analysis are encouraging, as they suggest that the subsample who are less likely to quit and who smoke more benefit the most from NEXit. The results also suggest that older adults in Sweden are willing to engage with digital health interventions. This finding contrasts with evidence that suggests a “digital divide” exists between older and younger adults in terms of acceptance and uptake of digital health interventions [31]. The current findings suggest that SMS text message–based interventions may be particularly apt for older adults.

### Confidence and Know-How

Baseline confidence was associated with individualized effects for both prolonged and point prevalence abstinence, suggesting that those who had greater confidence in their ability to quit benefited more from NEXit. This finding supports extant literature that highlights higher rates of self-efficacy (ie, confidence) are associated with a greater likelihood of successful smoking cessation [32-34]. The results for know-how varied, with no association found for the first time point for both prolonged and point prevalence abstinence; however, an association was found at the second time point for both outcome measures, with those displaying a higher rating of know-how benefiting more. It may be the case that participants had been lacking knowledge on how to quit and maintain abstinence over a short period of time, while they may have had knowledge on how to sustain abstinence over a longer period. This suggests that NEXit may be beneficial to those who are unsure of how to initially stop smoking and maintain the initial phase of abstinence.

### Gender

Associations between both importance and gender with individualized effects were found, with those who deemed it of greater importance to quit benefiting more, while effects were more pronounced for men compared to women. The finding for importance supports extant literature that has assessed motivation to quit (ie, the reasons smokers consider it important to quit), which suggests that higher motivation increases the likelihood of cessation and predicts abstinence [34-37]. Results compared across effectiveness trials highlight that women are less likely to remain abstinent than men [22], and the findings of the current study support this suggestion. Nonetheless, the association between effect and gender dissipated when adding the interaction term of gender and importance to the model, suggesting that, between men and women who deemed it of greater importance to quit, the effects of NEXit were greater for men. Future studies should consider exploring why importance may not be a driving force for change among women but is so for men. It should be noted that the sample included more women than men, reflecting greater treatment seeking among women. The parent trial was pragmatic in the sense that it was an effectiveness trial recruiting participants in a way that mimicked how a real-world implementation could work (ie, online advertisements). Therefore, our findings should most conservatively be generalized to those seeking help online, and not the general population, where the prevalence of smoking is more balanced.

On the other hand, for point prevalence, there were no marked associations between effects or interactions with respect to gender. This suggests that rates of relapse (or lack of) may have been similar between women and men, which is in contrast to evidence suggesting that women are more likely to relapse than men [21]. The content for craving provided by NEXit may have been equally effective for the needs of both genders; evidence suggests that affective factors such as mood, anger, and perceived stress predict relapse in women, while the motivation to reduce craving is a unique relapse predictor in men [38]. The support for craving had specific content relating to stress management, reframing perspectives, and motivation.

The results highlight that confidence and importance (especially for men) are potential focal considerations for smoking cessation. Confidence and importance are integral parts of the decision to quit smoking, especially for individuals who are contemplating or preparing for a change and who may feel ambivalent about quitting [39]. It may be that those who reported the importance of quitting and their confidence as high were better psychologically prepared to change behavior. This suggests that future interventions should target improvements in both factors.

### Smoking and Number of Quit Attempts

Associations between the number of cigarettes smoked and years of smoking with individualized effects were found for both prolonged and point prevalence abstinence, with those who smoked less and for a shorter duration benefiting the most. This suggests that NEXit is less beneficial for those who smoke more and have done so for a longer duration. Theories of addiction posit that when an addiction progresses, such as nicotine dependence, control over consumption shifts from goal-directed behavior to automatic or habitual motivation [40], hence those who smoke more and for longer *may* have been driven more by habit than goal-directed behavior. While the content of NEXit did include advice on how to avoid smoking cues, this may not have been enough for these heavier, longer-term smokers. Providing greater support for identifying what triggers habitual smoking and advice on how to plan for encountering triggers or cues may be beyond the scope of an SMS text message–based intervention. Potentially, this could be better achieved by smoking cessation counseling or through the usage of an interactive digital smoking cessation intervention, whereby end users are encouraged to record how they are triggered, along with creating their own plans to overcome their triggers, which can then be sent as reminders.

For prolonged abstinence, an association between quit attempts and individualized effect was found at 8 weeks, with those having fewer attempts at baseline benefiting more. The underlying action of NEXit may account for this finding, with the intervention theorized to be driven by instilling confidence in ability and raising the importance of quitting. Evidence highlights that self-efficacy (ie, confidence) and motivation (ie, importance) are key factors in smoking cessation for individuals who are less likely to make a quit attempt [37]. It may be that the mediating effects of confidence and importance on prolonged abstinence had a greater impact on this group than on those with greater recorded quit attempts at baseline. Nonetheless, at the 5-month follow-up, an individualized effect on prolonged abstinence was found for those reporting more quit attempts at baseline. This effect is potentially driven by these individuals building more confidence and gaining knowledge for quitting over the course of the trial.

### Degree of Dependence

Finally, for the dependence score, only an association with individualized effect for point prevalence at 3 months was found, with those reporting less dependence benefiting more. No other associations with dependence were observed. Dependence as a factor in cessation and abstinence is well established; the likelihood of quitting is much greater at low levels of dependence, in comparison to high levels [41]. The disparity in our findings with the literature may be due to the methods used to assess dependency. We used a self-report measure, whereas many studies use biochemical measures of dependence [42]. While the Fagerström scale has been shown to predict abstinence across trials [24], self-report measures are more prone to biases in comparison to objective measures such as a saliva cotinine test. While future studies should consider both self-reports and biochemical measures, it may not be feasible for the digital modality. Furthermore, evidence highlights selection biases present in saliva testing [43], while recommendations suggest self-report measures are more appropriate for general population studies [43]. Nonetheless, our results for dependence could be interpreted to mean that, by and large, NEXit is equally effective regardless of dependence severity.

### Limitations

The results provide insight into the individualized effects of the recent NEXit trial; however, they should be interpreted with some considerations. Typically in RCTs, the randomized component is tested, rather than a comparison across treatments, meaning individuals may adhere to or engage in an intervention at their own preference. Therefore, the results should be considered in terms of having access to the intervention rather than using it. Indeed, the aim of the trial was to estimate the effectiveness of providing a digital smoking cessation intervention; therefore, all intercurrent events were acceptable. We do note that since we did not collect data on the use of other interventions throughout the trial period, we cannot investigate if the NEXit intervention increased the utilization of other treatments.

Another limitation of this study is that a statistical model was used to predict and contrast outcomes. This means that the estimated effects are not based entirely on observed data but on predicted data. These predictions may have considerable uncertainty that is not readily quantifiable, as no individuals received both conditions; thus, no individual contrasts exist against which predictions can be compared. The result of this is that when comparing individualized effects against baseline factors, there is uncertainty that is not accounted for. More research is needed to develop methods to estimate the uncertainty of the statistical approach taken [27].

It should be noted that the relationships between baseline characteristics and individualized effects are associational and should not be interpreted as causal. Confounding and collider bias can substantially alter estimated associations. Further, the current study investigated several associations between baseline characteristics and individualized effects. The Bayesian inference paradigm informs which associations are stronger and weaker relative to one another and which associations have the relatively strongest evidence, conditional on the data and prior knowledge. By using Student *t* test priors centered at 0, there was weak shrinkage induced in these estimations to protect against overestimation. Thus, the result of this study is all the associations presented and not only those that pass a prespecified threshold of significance (eg, null-hypothesis testing). In all, the findings presented in this study should be seen as exploratory, and future studies should look to confirm the associations as causal.

### Conclusions

In conclusion, the results of this study highlight that individuals participating in the RCT of the NEXit intervention had heterogeneous outcomes, which were not discernible from the primary analysis of the RCT that compared the intervention and control groups [11]. The intervention was most effective among men, older individuals, those who smoked less at baseline and for a shorter duration, and those who had confidence in their ability and deemed it important to quit. The results have the potential to help refine smoking cessation interventions so that we can target those who persist in smoking despite current intervention efforts and policy measures.

## Supplementary material

10.2196/63578 Checklist 1 CONSORT checklist.

## Abbreviations

CoI: compatibility interval
CONSORT: Consolidated Standards of Reporting Trials
NEXit: Nicotine Exit
OR: odds ratio
RCT: randomized controlled trial
